# Tetanus Traumaticus Cured by Oil of Turpentine

**Published:** 1831-04-01

**Authors:** 


### III. TETANUS TRAUMATICUS CURED BY OIL OF TURPENTINE.

DR. GIBBON (of Swansea) has reported a case of this kind in our cotemporary, the GAZETTE. The patient was a female who received a wound in her hand, and in ten or twelve days afterwards shewed symptoms of tetanus, and this disease soon became established. Dr. G. ordered a liniment of oil of turpentine and laudanum to be rubbed over the greater part of the body. An injection, composed of two ounces of oil of turpentine, one ounce of olive oil, with gruel, was administered, and six grains of calomel were ordered every six hours. These medicines were continued for several days, with some modifications, and in eight or nine days the disease might be said to be subdued. There was then slight ptyalism established.

